# Quantitative Mapping of Cocaine-Induced ΔFosB Expression in the Striatum of Male and Female Rats

**DOI:** 10.1371/journal.pone.0021783

**Published:** 2011-07-01

**Authors:** Satoru M. Sato, Anne Marie Wissman, Andrew F. McCollum, Catherine S. Woolley

**Affiliations:** Department of Neurobiology and Physiology, Northwestern University, Evanston, Illinois, United States of America; The Research Center of Neurobiology-Neurophysiology of Marseille, France

## Abstract

ΔFosB plays a critical role in drug-induced long-term changes in the brain. In the current study, we evaluated locomotor activity in male and female rats treated with saline or cocaine for 2 weeks and quantitatively mapped ΔFosB expression in the dorsal striatum and nucleus accumbens of each animal by using an anti-FosB antibody that recognizes ΔFosB isoforms preferentially. Behavioral analysis showed that while there was little difference between males and females that sensitized to cocaine, nonsensitizing rats showed a large sex difference. Nonsensitizing males showed low behavioral activation in response to cocaine on the first day of treatment, and their activity remained low. In contrast, nonsensitizing females showed high activation on the first day of treatment and their activity remained high. Western blot and immunohistochemical analyses indicated that basal levels of ΔFosB were higher in the nucleus accumbens than the dorsal striatum, but that the effect of cocaine on ΔFosB was greater in the dorsal striatum. Immunostaining showed that the effect of cocaine in both the dorsal striatum and nucleus accumbens was primarily to increase the intensity of ΔFosB immunoreactivity in individual neurons, rather than to increase the number of cells that express ΔFosB. Detailed mapping of ΔFosB-labeled nuclei showed that basal ΔFosB levels were highest in the medial portion of the dorsal striatum and dorsomedial accumbens, particularly adjacent to the lateral ventricle, whereas the cocaine-induced increase in ΔFosB was most pronounced in the lateral dorsal striatum, where basal ΔFosB expression was lowest. Sex differences in ΔFosB expression were small and independent of cocaine treatment. We discuss implications of the sex difference in locomotor activation and regionally-specific ΔFosB induction by cocaine.

## Introduction

Drug abuse gives rise to long-term changes in brain function. ΔFosB, a truncated and long-lasting form of the immediate-early gene FosB, has been studied extensively as a mediator of long-term structural and functional changes within the reward circuitry of the brain, especially in response to psychostimulants [1]. For example, overexpression of ΔFosB enhances sensitivity to psychostimulants, evidenced by heightened locomotor responses to cocaine [2] and decreased threshold for conditioned place preference[2] and self-administration [3].

ΔFosB accumulation has been most studied in the rodent striatum [4]. The striatum is generally divided into dorsal striatum (DS, caudate-putamen) and ventral nucleus accumbens (NAc). The nucleus accumbens is further divided into core and shell, based on chemorachitecture [5], [6], [7] and function [8]. The principal cells in the striatum are GABAergic medium spiny neurons (MSNs) [9]. Subtypes of MSNs are classified based on their projections and chemoarchitecture. Striatonigral MSNs express D1 dopamine receptors, Substance P and dynorphin (Dyn), whereas striatopallidal cells express mostly D2 receptors and enkephalin. These classes of MSNs are largely distinct in the dorsal striatum [10], [11], but less so in the NAc [12]. ΔFosB is preferentially, but not exclusively, expressed in striatonigral projection cells [13], [14]. In addition, ΔFosB accumulation varies subregionally with different drugs [15] and over time during the course of drug administration and withdrawal [13].

Behavioral manifestations of drug abuse are sexually dimorphic, and women may be more vulnerable to drug abuse [reviewed in 16,17,18]. In preclinical studies with rodents, females show greater locomotor responses [19], [20], [21], [22], motivation to take drug [23], [24], and preference for drugs [25] than males do. Sex differences in physiology [26], [27] and anatomy [28] of MSNs, as well as in striatal dopamine responses to drugs [reviewed in 29] likely underlie these behavioral differences, although sex differences are not fully understood.

While drug-induced ΔFosB expression has been described [15], [30], [31], detailed quantitative investigation of drug-induced ΔFosB accumulation among striatal suregions in males versus females has been lacking. Psychostimulants are known to alter immediate-early gene expression in regionally specific manners [see 32 for review] and amphetamine-induced c-*fos* expression is sexually dimorphic and regionally specific [33]. Given the important role of ΔFosB in the effects of psychostimulants on synaptic input to MSNs [34], more detailed description of ΔFosB accumulation in both sexes could aid in determining how subregional variation in the neural effects of drugs relate to drug addiction. In the current study, we quantified the effects of repeated cocaine administration on locomotor behavior and FosB immunoreactivity in the DS and NAc of male and female rats. We used an antibody selective for ΔFosB and unbiased stereology to obtain anatomically detailed and quantitative measures of cocaine-induced ΔFosB accumulation.

## Methods

### Ethics Statement

All animal procedures were performed in accordance with the National Institutes of Health *Guide for the Care and Use of Laboratory Animals* and were approved by the Northwestern University Institutional Animal Care and Use Committee (protocol 2009-0969). All efforts were made to minimize suffering of animals used for this study, and animals were euthanized with an overdose of sodium pentobarbital.

### Animals

Gonadally intact young adult male and female Sprague-Dawley rats (Harlan, Indianapolis, IN), ∼50 days old at the start of experiments, were used. Animals were housed in same-sex groups of 2 or 3 per cage and kept under a 12∶12 light:dark cycle. Soy-free rat chow (Teklad #2916, Harlan, Indianapolis, IN) and water were available ad libitum. Estrous cycles in females were not monitored to avoid vaginal lavage-induced stress, since stress is known to induce ΔFosB accumulation in many brain areas [35].

### Cocaine Treatment and Behavioral Monitoring

All behavioral testing was conducted during the mid-late light phase. On each treatment/testing day, rats were placed individually in 45 cm ×45 cm ×45 cm testing chambers, and allowed to acclimatize for 30 min. Each rat was then injected (i.p.) with saline or cocaine (15 mg/kg), immediately returned to the chamber, and allowed to move freely for 60 min after injection. Locomotor behavior was digitally recorded using LimeLight software (Actimetrics, Wilmette, IL) during the 30 min acclimation period and then for 60 min following injection. Data are expressed as mean±SEM cumulative distance traveled during the 60 min post-treatment period. Rats were treated for 2 weeks using a 5 days on 2 days off treatment regimen, and then sacrificed 24 hrs after their last injection to evaluate FosB protein levels by western blot or immunohistochemistry (IHC) for FosB immunoreactivity (FosB-IR) in the dorsal striatum (DS) and nucleus accumbens (NAc). Rats were kept in their regular housing facility until immediately before sacrifice.

### Western Blots

Male and female rats (n = 3 per group) were deeply anesthetized with sodium pentobarbitol (80 mg/kg, i.p., Virbac Animal Health, Fort Worth, TX) and transcardially perfused with ice-cold HEPES buffer (10 mM, with 0.5 mM phenylmethanesulfonylfluoride (PMSF), 1 µg/ml leupeptin and 1 µg/ml aprotinin). Brains were removed and the DS and NAc were dissected, homogenized in cold buffer (20 mM HEPES, 20% glycerol, 1% NP-40, 5 mM dithiothreitol 0.5 mM PMSF, 10 µg/ml leupeptin and 1 µg/ml aprotinin), and stored at −80°C. Protein assays were performed and samples of equivalent total protein were prepared in Laemmli sample buffer. Samples were run on 12% SDS gels and transferred to PVDF membranes. Membranes were blocked in 5% nonfat milk in PBS and incubated with each of 3 anti-FosB primary antibodies [sc-48 (1∶1,000, Santa Cruz Biotechnology, Santa Cruz, CA), H-75 (1∶1,000, Santa Cruz Biotechnology), and 5G4 (1∶2,000, Cell Signaling Technology, Danvers, MA)] overnight at 4°C. After primary antibody incubation, membranes were blocked again in milk and incubated with peroxidase-conjugated secondary antibodies (goat anti-rabbit, 1∶1,000, Vector Laboratories, Burlingame, CA) and visualized with ECL-Plus chemiluminescence (GE Health Care, Piscataway, NJ). After exposure to film (GE Hyperfilm), blots were stripped and reprobed for actin (I-19, 1∶2,000, Santa Cruz Biotechnology) as a loading control.

Because 5G4 recognized primarily lower molecular weight bands (34–37 kD) characteristic of ΔFosB, 5G4 was used subsequently to evaluate ΔFosB protein levels in cocaine- or saline-treated male and female rats by western blot and IHC. For western blots, ImagePro *Plus* v.6.2 software (MediaCybernetics, Bethesda, MD) was used to measure optical density of bands in the 34–37 kD range, and this was normalized to actin. All samples were run in duplicate and the optical density values for each animal were averaged for statistical analysis.

### FosB immunohistochemistry

For analysis of FosB IHC in the DS, we used 9 rats per group. For the NAc, an additional 6 rats per group were added for a total of 15 rats per group. Twenty four hrs after the last cocaine or saline injection, rats were deeply anesthetized with sodium pentobarbitol (80 mg/kg, i.p., Virbac Animal Health) and transcardially perfused with 4% paraformyladehyde in 0.1 M phosphate buffer (PB, pH 7.4). Brains were removed, post-fixed in the same fixative overnight at 4°C, cryoprotected in 30% sucrose in PB, and then 50 µm coronal sections through the NAc and DS were collected into 5 series. Brain sections were stored in 10% sucrose and 0.03% sodium azide in PB at 4°C until further processing.

FosB IHC was carried out at room temperature unless otherwise stated. Every 5^th^ section was initially treated with warm (∼80°C) 10 mM citric acid buffer (pH 6.0) for 30 min to retrieve antigen [36]. Subsequently, the sections were immuno-stained with the 5G4 anti-FosB antibody (1∶400, Cell Signaling Technologies) followed by 3,3’-diaminobenzidine (DAB) visualization using standard protocols [37]. Briefly, sections were rinsed 2×10 min in PB, incubated in freshly prepared 1% sodium borohydrate in PB for 10 min, rinsed 3×5 min in PB, 2×5 min in 0.1 M Tris-buffer (TB, pH 7.4), followed by incubation in 0.05% hydrogen peroxide in TB for 30 min, 0.1% hydrogen peroxide in TB for 60 min, 0.05% hydrogen peroxide in TB for 30 min, rinsed 2×5 min in TB, and 3×10 min in 0.1 M Tris-buffered saline (TBS, pH 7.4). Subsequently, sections were incubated in blocking solution containing 5% normal goat serum (NGS, Vector Laboratories), 3% bovine serum albumin (BSA), 0.3% dimethyl sulfoxide (DMSO) in 0.5 M TBS for 60 min. Following 9×5 min rinses in TBS, sections were incubated in anti-FosB antibody diluted in 0.5 M TBS containing 1% NGS, 2% BSA, 0.3% DMSO for ∼36 hrs at 4°C. Following 9×10 min rinses in TBS, sections were incubated in biotinylated anti-rabbit secondary antibody (1∶800, Vector Laboratories) for 3 hrs, rinsed 9×10 min in TBS, followed by rinses in 2×10 min 0.1 M TBS, 2×5 min in TB (pH 7.4), and 2×5 min in TB (pH 7.6). Immunoreactivity was visualized by incubation in 0.025% DAB and 0.01% hydrogen peroxide in TB (pH 7.6) for 5 min. Sections were then rinsed 2×5 min in TB (pH 7.6), 2×5 min in TB (pH 7.4), 1×5 min in PB, mounted on gelatin-coated slides, air-dried, counterstained with cresyl violet, dehydrated in ascending series of alcohol, cleared in xylene, and coverslipped with Eukitt Mounting Medium (Electron Microscopy Sciences, Hatfield, MA).

### Quantification of FosB-IR

Slides were coded prior to analysis. The proportions of all neuron-like cells, identified by cresyl violet staining, in the DS and the NAc containing FosB-IR nuclei were estimated using random-systematic sampling and the optical fractionator probe [38], [39]. Tissue was visualized with an Olympus BX60 microscope (Olympus America, Center Valley, PA) equipped with StereoInvestigator 9 system (MicroBrightField, Williston, VT), including a CCD camera and a XYZ-motorized stage. The DS, NAc Core, and NAc Shell were delineated under a 4x objective and cells were counted under a 100x oil-immersion objective. Throughout the study, 30 µm×30 µm×8 µm counting frames were used. For the DS, labeled nuclei were counted random-systematically in counting frames distributed as 500 µm×500 µm grids in 3 sections (anterior-posterior (AP): +1.56–0.60 mm relative to Bregma), for approximately 208 counting frames (1.50×10^6^ µm^3^) per animal. For the NAc, 350 µm ×350 µm grids were used in 4 sections (AP: +2.40 – 1.20 mm relative to Bregma) for approximately 191 counting frames (1.38×10^6^ µm^3^) per animal. The grid size was determined through a pilot study to achieve a stereologically reliable sampling density (Coefficient of Error<0.1) [40]. The intensity of staining in each labeled nucleus was categorized as either light, medium, or dark (see Fig. 3 for examples). Light staining was defined as light DAB reaction product clearly visible in the nucleus without obscuring the view of the nucleolus, medium staining was defined as DAB reaction product partially obscuring the view of the nucleolus, and dark staining was defined as DAB reaction product completely obscuring the view of the nucleolus. FosB-IR data are expressed as mean±SEM percent of all neuron-like cells. To verify the consistency of staining intensity classification, random-systematically sampled cells from a subset of animals in each group were analyzed for their mean optical density using ImageJ. There were no significant treatment- or sex-related differences in measured optical density of nuclei within an intensity class, and each class of cells (light, medium, or dark) was separated by approximately 20 optical density units on 8-bit gray scale (0–256), on average.

**Figure 1:**
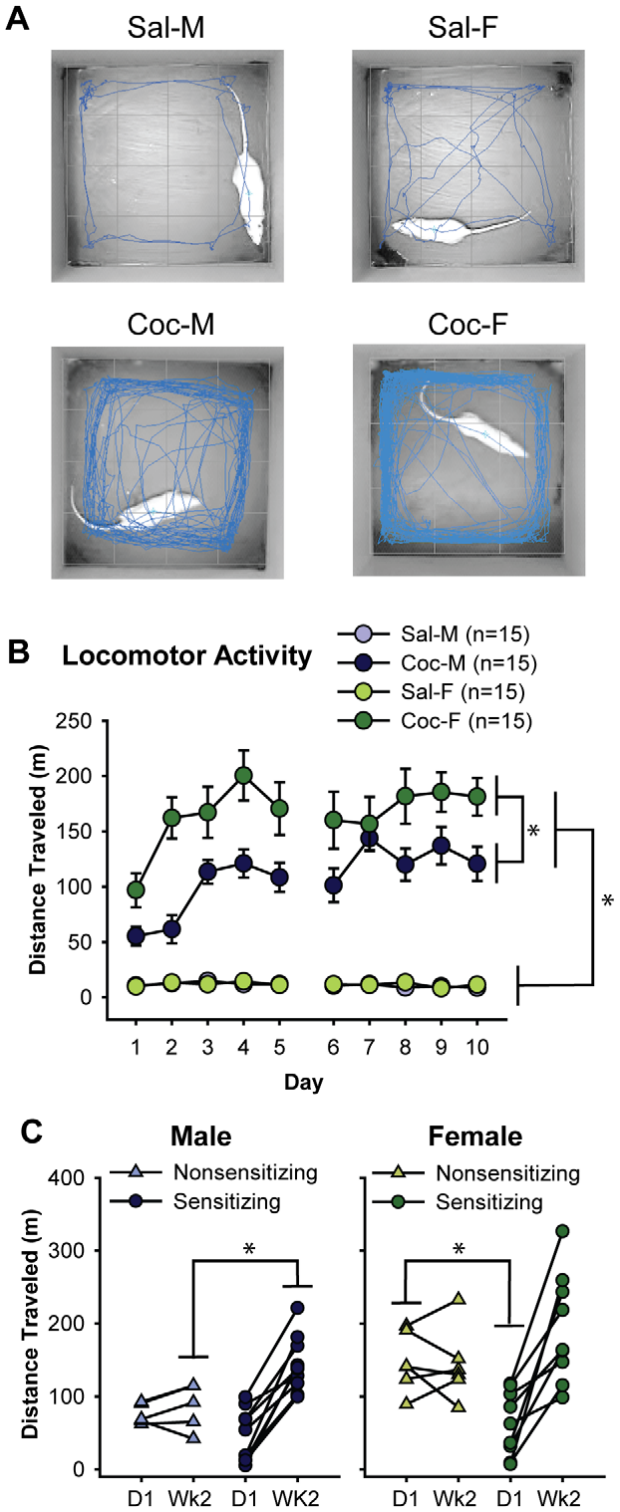
Cocaine increased locomotor activity to a greater extent in females than in males. A) Representative locomotor activity traces during the initial 5 min following saline (Sal) or cocaine (Coc) injection on the last day (Day 10) of treatment in males (M) and females (F). Blue lines represent the movement of each animal during this period. B) The mean±SEM distance traveled during 60 min following cocaine or saline injection each day over the 2 week treatment period. Cocaine increased locomotion, especially in female rats. C) Distance traveled on the first day (D1) and the average of week 2 (Wk2) for cocaine-treated male (left) and female (right) rats. Data are displayed separately for sensitizing and nonsensitizing rats; connected points represent data for the same individual. Nonsensitizing males showed consistently low activity throughout treatment, whereas nonsensitizing females showed consistently high activity throughout treatment. *p<0.05.

### Anatomical partitioning of dorsal striatum and nucleus accumbens

To evaluate subregional variation in FosB staining, the DS was divided into 4 zones, similar to the scheme used by Steiner and Gerfen ([41] Fig. 4C]. The DS ventral to the ventral limit of the lateral ventricle was classified as Ventral (V), and the remaining DS was divided into 3 zones of equal medial-lateral width for Medial (M), Intermediate (I), and Lateral (L) zones (Fig. 4C). Similarly, the NAc Core was divided into 4 quadrants and 2 anterior (A) and 2 posterior (P) sections collapsed for 8 final subdivisions (4 quadrants×2 AP levels, Fig. 6A). As such, both the anterior (A) and posterior (P) NAc Core was divided into dorsomedial (DM), dorsolateral (DL), ventromedial (VM), and ventrolateral (VL) quadrants (Fig. 6A). The NAc Shell was divided into 2 halves and 2 anterior and 2 posterior sections collapsed for 4 final subdivisions (2 halves×2 AP levels, Fig. 7A). Both the anterior and posterior Shell was divided into dorsomedial (DM) and ventrolateral (VL) halves (Fig. 7A). Each zone was named based on AP, dorsal-ventral (DV), and medial-lateral (ML) location within a structure, so that the dorsomedial quadrant in the anterior NAc Core was named ADM, for example (see Figs. 4C, 6A, and 7A for details).

**Figure 2:**
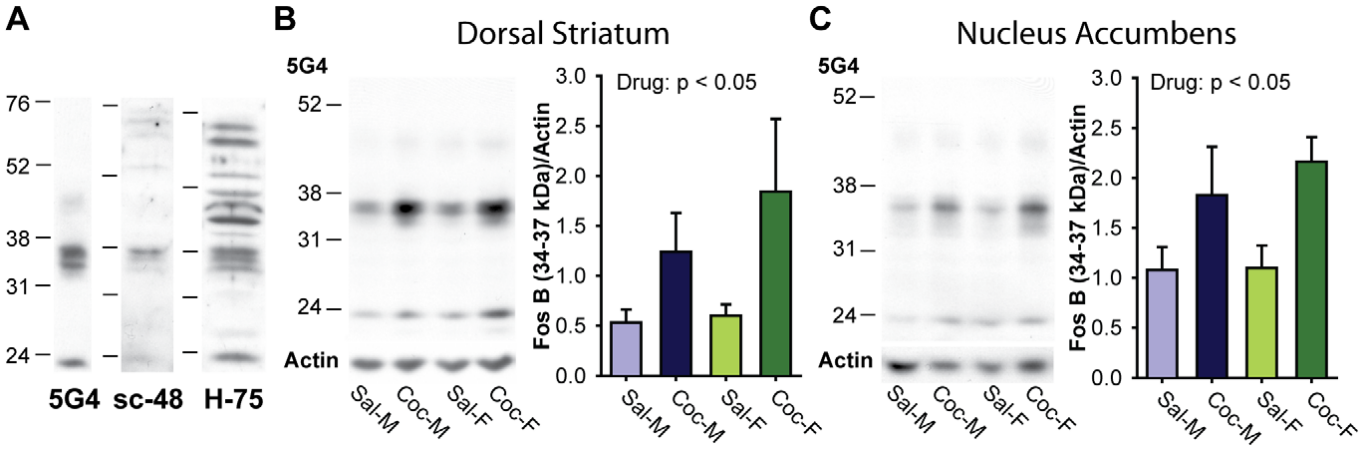
Cocaine increased ΔFosB protein levels in the dorsal striatum and nucleus accumbens. A) Representative western blots with three FosB antibodies: 5G4, sc-48, H-75. Due to its specific and robust labeling for ΔFosB isoforms (34–37 kD), 5G4 was used for subsequent experiments. B) Representative western blot of dorsal striatum samples from 2-week cocaine- or saline-treated males and females probed with 5G4 anti-FosB. The same blot reprobed for actin is shown below. Bar graph shows quantification of optical density from n = 3 animals/group. C) Representative western blot of nucleus accumbens samples from 2-week cocaine- or saline-treated rats probed with 5G4 anti-FosB. The same blot reprobed for actin is shown below. Quantification of optical density from n = 3 samples/group. Two-way ANOVAs showed that cocaine significantly increased ΔFosB in both dorsal striatum (B) and nucleus accumbens (C).

**Figure 3:**
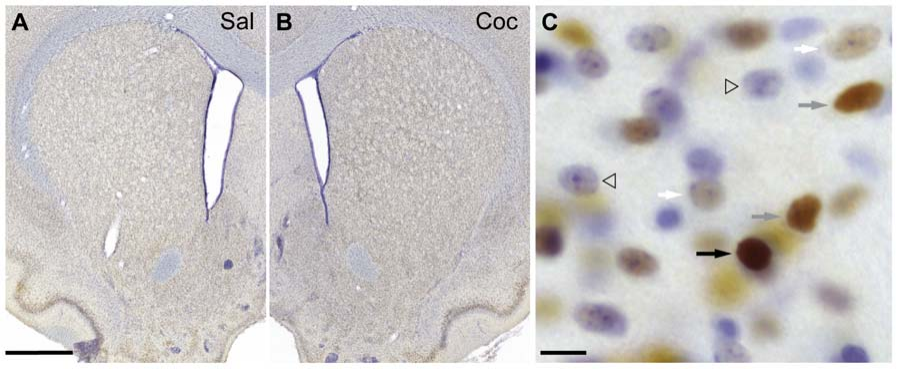
FosB immuno-staining in the striatum. Representative low magnification photomicrographs of striatal sections immuno-stained for FosB (5G4) from a saline- (A) and cocaine- (B) treated rat; sections were counter-stained with cresyl violet. C) Higher magnification photomicrograph from the dorsal striatum of a cocaine treated rat. FosB-IR nuclei of varying staining intensities that are in focus are indicated by arrows (dark = black arrow, medium = gray arrow, and light = white arrow); several cresyl violet-stained cells that are FosB-negative are indicated by open triangles. Scale bars: 100 µm for A and B, 10 µm for C.

**Figure 4:**
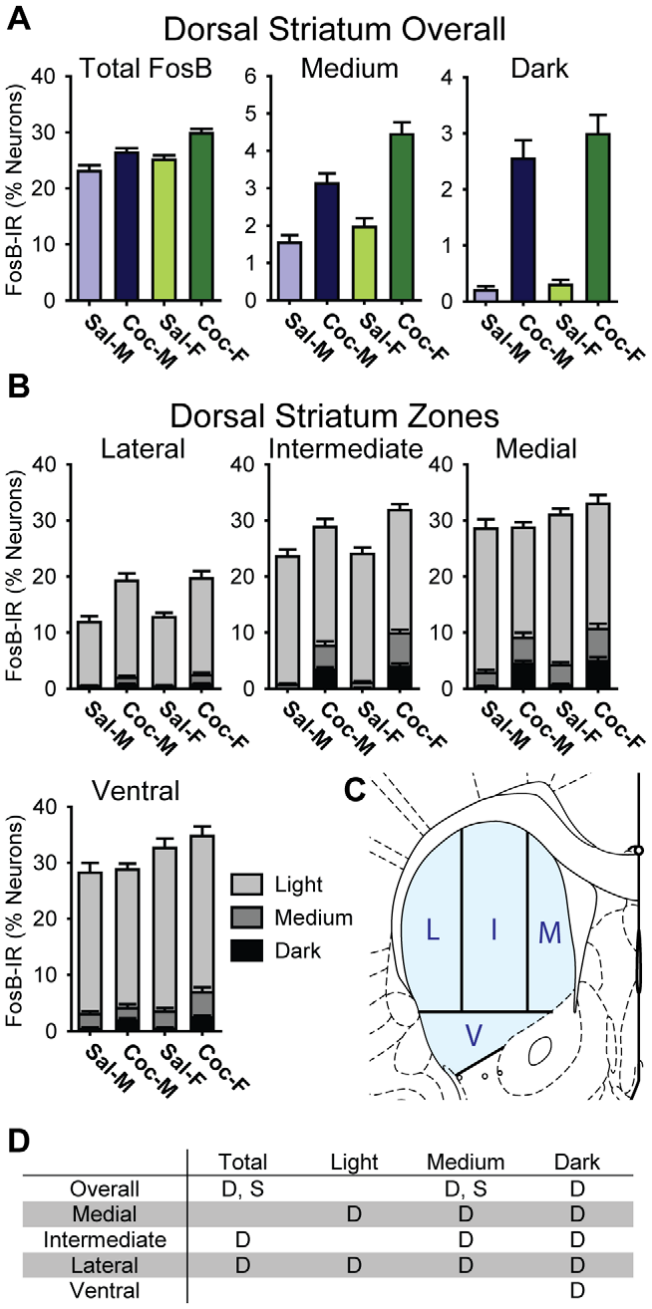
Cocaine induced regionally specific increases in FosB- IR in the dorsal striatum of male and female rats. A) Bar graphs showing the percentage of all neurons in saline- (Sal) and cocaine- (Coc) treated male (M) and female (F) rats that were FosB-IR at any intensity level (Total FosB), and broken down into medium or darkly stained cells. B) Bar graphs showing the proportion of FosB-IR cells in each anatomical zone of the dorsal striatum. Each bar shows the percentage of all neurons in each treatment group that were light, medium, or darkly stained. Overall FosB-IR was higher in the Medial and Ventral, relative to the Lateral, zone, while cocaine-induced increases were most prominent in the Lateral zone. Additionally, females exhibited slightly higher FosB-IR than males. C) The partitioning scheme used to define Medial (M), Intermediate (I), Lateral (L), and Ventral (V) zones. D) A table summarizing the statistically significant differences (p<0.05) for dorsal striatum as a whole (Overall) and each of the zones. “D” indicates a significant drug effect, and “S” indicates a significant sex difference (See text).

### Heat Maps

In order to display patterns of FosB-IR, heat maps were created from a subset of sections from the rats included in the analyses of both DS and NAc (9 animals per group). To create the heat maps, the xy-coordinate of each counted cell, as well as the coordinates of the lateral ventricles and the anterior commissure, were exported from StereoInvestigator and plotted in SigmaPlot 11 (Systat Software Inc. San Jose, CA). The plot of each section was sorted according to its rostrocaudal coordinates, aligned in Adobe Illustrator CS4 (Adobe Systems, Inc. San Jose, CA) using the lateral ventricle and the anterior commissure as references. A marker representing each counted cell was resized to roughly fill the counting grid that contained the respective counting frame. The markers were converted to gray scale based on their classification (dark: 60, medium: 30, light: 5, in inverted 8-bit gray scale). The generated vector graphics were converted to gray scale raster images, smoothed by applying Gaussian blur filter (sigma: DS: 30 px and NAc: 40 px), a color lookup table was applied in ImageJ (NIH, Bethesda, MD), and heat maps were superimposed on panels from the rat brain atlas [42]. Each map was generated from 12 hemispheres per rostrocaudal level, chosen on the basis of best fit with the respective panel of the brain atlas and representing 2/3 of all sections analyzed for FosB-IR.

### Statistical Analysis

Locomotor Behavior: The 60 rats used for FosB-IHC were analyzed for locomotor behavior. Distance traveled on each of 10 injection days was analyzed with 3-way ANOVA (sex×drug×day). In addition, distance traveled on the first day of injection (D1), the average distance traveled in the 2^nd^ week (Wk2, days 6–10), and the ratio of Wk2 to D1 (Wk2/D1) were analyzed with 2-way ANOVAs (sex×drug). Cocaine-treated males and females were categorized as showing behavioral sensitization according to criteria established by Boudreau and Wolf [43]. A cocaine-treated animal was considered to show sensitization if its Wk2/D1 ratio exceeded the average C.V. for the saline animals. For additional analyses of locomotor sensitization, Wk2 and D1 data were compared between sensitizing and nonsensitizing rats with unpaired Student’s t-tests. Significant interactions and main effects in ANOVAs were further analyzed with appropriate lower order ANOVAs and Student’s t-tests.

FosB-IR: For analysis of FosB protein levels with western blots, a 2-way ANOVA (sex×drug) was used, followed by unpaired Student’s t-tests. For analyses of FosB-IR nuclei with IHC, we used 4-way mixed ANCOVAs (sex×drug×AP level×zone) for the DS and the NAc shell. For the NAc core, we used a 5-way mixed ANCOVA, where zones were substituted by dorsal-ventral and medial-lateral levels. Owing to the large number of animals in this study, we processed sections in two cohorts with an equal number of animals from each group. As such, cohort number was included as a covariate to account for any differences between cohorts. Significant interactions and main effects were further analyzed with appropriate lower-order ANCOVAs and/or Student’s t-tests. Separately, the data from cocaine-treated animals were analyzed as above with the sensitization classification substituted for drug treatment as an independent variable.

Bonferroni correction was used for all multiple comparisons with t-tests. All statistical analyses were conducted with PASW Statistics 18 (SPSS, Inc., Chicago, IL), and p<0.05 was considered significant.

## Results

### Cocaine-induced locomotor behavior in male and female rats

#### Overall Locomotor Behavior

The expected cocaine-induced increase in locomotion is apparent in Fig. 1A, which shows representative tracking data from each of the 4 groups studied. The blue lines represent locomotion during the first 5 min after saline or cocaine injection on the last treatment day.

Over 10 days of drug treatment, cocaine significantly increased locomotor activity (Fig. 1B, drug: F_1,56_ = 206.5, p<0.001). Upon the initial injection (D1), locomotor activity was significantly greater in cocaine- than saline-treated rats (saline vs. cocaine: F_1,56_ = 55.6, p<0.001); responses to cocaine increased during the first week and remained high during the second week (Wk2: F_1,56_ = 171.5, p<0.001). Averaged across the treatment period, cocaine-treated females showed significantly more locomotor activity than cocaine-treated males (males: 108.2±6.9 m/day vs. females: 166.2±16.0 m/day, F_1,28_ = 11.0, p = 0.003), while no sex difference was observed in saline-treated rats (males: 11.2±0.6 m/day vs. females: 11.7±1.0 m/day, F_1,28_ = 0.2, ns). Females’ greater locomotor responses to cocaine were apparent from the start of drug treatment, on D1 (males: 55.3±8.5 m/day vs. females: 96.8±15.3 m/day; t_28_ = 4.1, p = 0.03), and this sex difference persisted into Wk2 (males: 124.5±11.5 m/day vs. females: 173.1±17.6 m/day; t_28_ = 3.4, p = 0.03). No such sex-difference was observed in saline-treated rats (D1: t_28_ = 0.4; Wk2: t_28_ = 1.1, both ns). Locomotor responses to cocaine increased over the course of 2 weeks (Wk2/D1 ratio: F_1,56_ = 6.1, p = 0.02) in both males and females. And, while cocaine-induced locomotion was consistently higher in females than in males, the degree of increase in locomotion was nearly identical in both sexes (Wk2/D1 ratio: males: 5.2±1.8 vs. females: 5.1±2.4; F_1,56_ = 0.001, ns).

#### Sex-specific Patterns of Locomotor Sensitization

Among the cocaine-treated rats, similar proportions of males (10 out of 15) and females (9 out of 15) showed locomotor sensitization to cocaine as defined previously [43]. Interestingly, however, while the pattern of locomotor activity in sensitizing rats was similar between males and females (D1: t_17_ = 1.0, and Wk2: t_17_ = 1.8, respectively, both ns; Fig. 1C), the behavior of nonsensitizing rats showed a significant sex difference. Specifically, nonsensitizing males showed low activity on D1 and their activity remained low, whereas nonsensitizing females started high on D1 and their activity remained high. On D1, nonsensitizing males responded to cocaine similarly as sensitizing males but then failed to increase their responses over time, resulting in much lower responses during Wk2 (sensitizing vs. nonsensitizing males: F_1,13_ = 8.7, p = 0.011, Fig. 1C left). Nonsensitizing females, on the other hand, exhibited greater responses to cocaine on D1 than sensitizing females did (sensitizing vs. nonsensitizing females: F_1,13_ = 13.8, p = 0.003, Fig. 1C right), and their responses remained high. The difference between nonsensitizing males and females on D1 was statistically significant (t_9_ = 3.7, p = 0.005). Thus, the higher locomotor response seen in females as a whole (Fig. 1B) is largely attributable to the behavior of nonsensitizing, but not sensitizing, rats.

### Characterization of FosB antibodies

We investigated 3 commonly used anti-FosB antisera to determine which best recognized ΔFosB isoforms. As shown in Fig. 2A, 5G4 showed the most robust labeling of ΔFosB (MW: 34–37 kD) with only a very weak band for the full length FosB (MW: ∼48 kD). The sc-48 antibody also recognized ΔFosB, but labeling was weaker than with 5G4. The H-75 antibody recognized multiple higher molecular weight bands, including strong bands for the full length FosB. Particularly H-75 and 5G4 also recognized a smaller isoform (MW: ∼21 kD). Based on its robust labeling of ΔFosB, we performed subsequent western blot and immunohistochemical studies with 5G4.

**Figure 5:**
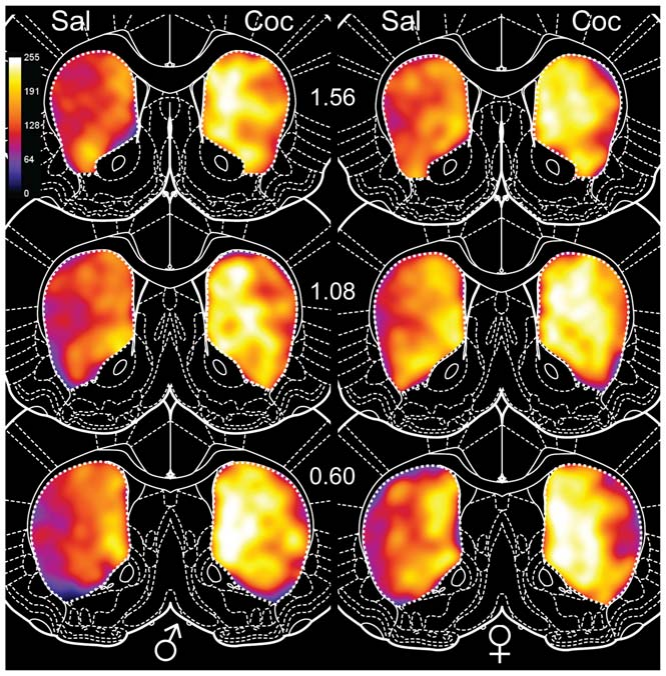
Heat maps of FosB-IR in the dorsal striatum. Numbers in the center indicate AP levels relative to Bregma. The cocaine-induced increase in FosB-IR is apparent at all levels in both male (left) and female (right) rats. Greater FosB-IR was seen in the medial, relative to the lateral, part of the dorsal striatum. Females exhibited higher FosB-IR than males, which is more apparent in the saline-treated rats. In addition, the cocaine-induced FosB-IR increase was larger in the more caudal (1.08 and 0.60) sections than in the rostral (1.56) section. Coc: cocaine, and Sal: saline.

**Figure 6:**
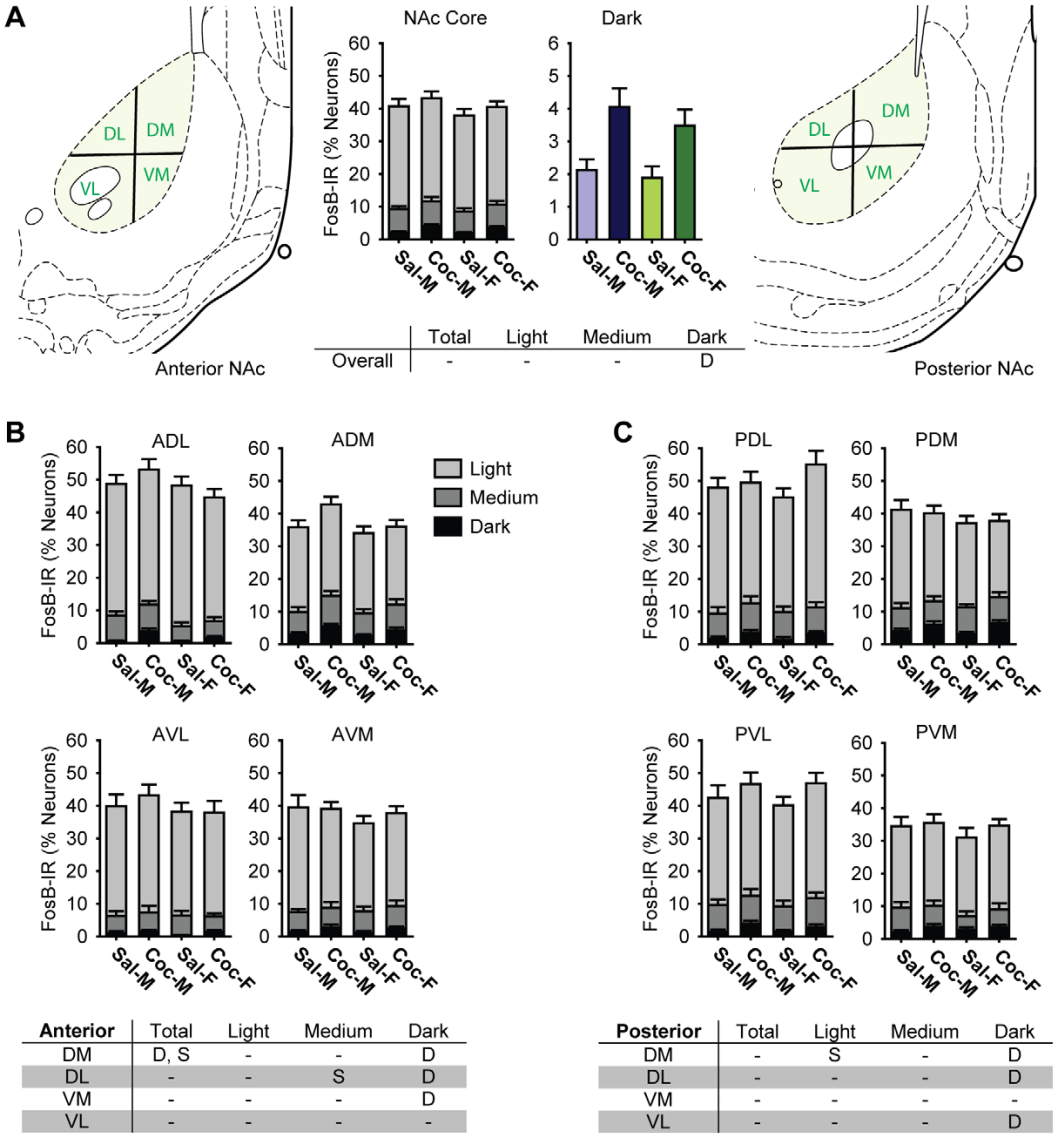
Cocaine selectively increased the proportions of neurons darkly stained for FosB in the nucleus accumbens (NAc) Core of male and female rats. A) Bar graphs showing the percentage of all neurons in saline- (Sal) and cocaine- (Coc) treated male (M) and female (F) rats that were FosB-IR. Each bar shows the percentage of all neurons in each treatment group that were light, medium, or darkly stained (middle left, NAc Core), and darkly stained cells only (middle right, Dark). The partitioning scheme used to define dorsal-ventral (DV) and medial-lateral (ML) zones for anterior (left) and posterior (right) NAc Core are also shown. B and C) Bar graphs showing the proportion of FosB-IR cells in each anatomical zone of the anterior (B) and posterior (C) NAc Core. Each bar shows the percentage of all neurons in each treatment group that were light, medium, or darkly stained. The tables summarize statistically significant differences (p<0.05) for the NAc Core as a whole (A), and in the anterior (B) and posterior (C) anatomical zones. “D” indicates a statistically significant drug effect, and “S” indicates a significant sex difference (See text).

### FosB protein levels in the Dorsal Striatum and Nucleus Accumbens

We used western blots as an initial approach to investigate cocaine effects on ΔFosB protein in the DS and NAc of males and females. As expected, cocaine treatment significantly increased ΔFosB protein in both regions. In the DS, cocaine increased ΔFosB levels by ∼170% (Fig. 2B, F_1,8_ = 8.2, p = 0.02). The effect of cocaine was slightly larger in females (208%) than males (133%), but there was no statistically significant sex difference (sex: F_1,8_ = 0.6, sex×drug: F_1,8_ = 0.4, both ns). In the NAc, cocaine increased ΔFosB levels to a lesser extent than in the DS, by ∼80% (Fig. 2C, F_1,8_ = 5.4, p = 0.049). As in the DS, the cocaine-induced increase in the NAc was slightly larger in females (97%) than in males (69%), but this was not a statistically significant difference (sex: F_1,8_ = 0.3, sex×drug: F_1,8_ = 0.3, both ns). Interestingly, based on the ΔFosB to actin ratio in both brain regions, basal ΔFosB levels in saline-treated animals were higher in the NAc than in the DS. This regional difference in basal ΔFosB levels and the greater effect of cocaine in the DS, were corroborated by subsequent immunohistochemical staining (see below).

Fig. 3 shows representative low magnification photomicrographs of brain sections from saline- (Fig. 3A) and cocaine-treated (Fig. 3B) rats stained for FosB using 5G4. Examples of FosB-negative cresyl violet-stained cells and FosB-positive cells with light, medium, or darkly stained nuclei are shown in Fig. 3C. From images like these, we quantified and mapped FosB-IR in the DS and NAc as the proportion of all neuron-like cells containing FosB-IR vs. non-IR nuclei and categorized each labeled nucleus as light, medium or dark. Overall, this analysis showed that the major effect of cocaine was to increase the intensity of FosB immunostaining, with relatively small effects on FosB-IR cell number.

### FosB-IR in the Dorsal Striatum

Cocaine treatment slightly, but significantly, increased the proportion of neuron-like cells in the DS that contained FosB-IR (saline: 24.1±1.1% vs. cocaine: 28.1±0.8%, F_1,31_ = 10.1, p = 0.003; Fig. 4A left). More prominently, cocaine increased the intensity of FosB-IR, in that cocaine’s effects in the DS were specific to medium and darkly-stained cells (medium: saline: 1.8±0.2% vs. cocaine: 3.8±0.3%, F_1,31_ = 54.9, p<0.001; dark: saline: 0.2±0.1% vs. cocaine: 2.8±0.3%, F_1,31_ = 63.8, p<0.001; Fig. 4A middle and right); cocaine did not significantly affect the number of lightly stained cells (light: saline: 22.1±1.0% vs. cocaine: 21.5±0.6%, F_1,31_ = 0.2, ns). The proportions of darkly stained cells showed the largest change, with over a ten-fold increase in cocaine-treated rats vs. saline-treated controls.

#### FosB-IR in the Dorsal Striatum Subregions

To investigate subregional differences in FosB labeling, we divided the DS into 4 zones: 3 dorsal and 1 ventral zone (Fig. 4C). This revealed significant differences between zones and a zone×drug interaction in all classes of FosB-IR cells (Fig. 4B). Statistically significant differences are summarized in Fig. 4D, with “D” indicating a drug effect and “S” indicating a sex difference for each class of cell in each zone. Post-hoc analyses revealed medial-lateral gradients in FosB-IR and in cocaine’s effects on FosB-IR. In general, the Medial, Intermediate, and Ventral zones contained more FosB-IR cells than the Lateral zone, and the effects of cocaine were greatest in the Lateral and Intermediate zones (Fig. 4B).

Specifically, in the Medial zone, the relative proportions of heavily stained (medium+dark) FosB-IR cells increased (+209%) with cocaine-treatment, and the proportion of lightly-stained FosB-IR cells decreased (−20%). As a result, the overall proportion of cells that were FosB-IR was unchanged in this zone. In contrast, in the Lateral zone, the proportions of all categories, light (+45%), medium (+293%), and dark (+2,262%) cells, increased with cocaine treatment. Consequently, the overall proportion of FosB-IR cells in the Lateral zone was significantly higher in cocaine-treated rats (saline: 12.3±0.9% vs. cocaine: 19.4±1.3%, F_1,31_ = 23.3, p<0.001). In the Intermediate zone, the pattern of FosB-IR combined features of the Medial and the Lateral zones. As was the case in the Medial and the Lateral zones, the proportions of heavily stained cells were significantly higher in cocaine-treated rats (medium: +517%, dark: +7,582%, relative to saline-controls). However, the proportion of lightly stained cells was unchanged. As a result, the total proportion of all cells that were FosB-IR was only modestly higher in the cocaine-treated rats. The pattern of FosB-IR and cocaine-induced changes in FosB-IR in the Ventral zone was similar to the Medial zone; the proportion of darkly stained cells increased (+410%) significantly with cocaine treatment, but the total proportion of all cells containing FosB-IR was unchanged.

In addition to these subregional differences in FosB-IR, we observed an anterior-posterior level x drug interaction in the proportion of heavily stained (medium+dark) cells (F_2,62_ = 5.5, p = 0.006). The cocaine-induced increase in heavily stained cells was more prominent in posterior DS sections. In the cocaine-treated rats, the proportion of heavily stained cells was significantly higher in the posterior sections, while such a difference was not seen in the saline-treated rats (saline: F_2,30_ = 0.1, ns; cocaine: F_2.30_ = 5.1, p = 0.01).

#### Sex Differences in the Dorsal Striatum Subregions

The proportion of neuron-like cells containing FosB-IR in the DS was slightly higher overall in females than in males (Fig. 4A, males: 24.7±1.1% vs. females: 27.4±1.0%, F_1,31_ = 4.7, p = 0.04). Broken down by staining intensity, this trend was observed in all 3 classes (light, medium, and dark) of FosB-IR cells, but statistically significant only in the medium stained cells (males: 2.3±0.3% vs. females: 3.2±0.4%, F_1,31_ = 10.1, p = 0.003). These sex differences were much smaller than cocaine-induced changes, however. For example, cocaine increased the proportion of darkly-stained cells by ∼1,000% overall compared to only a ∼20% difference between sexes. No significant sex differences were observed in subregional patterns of FosB-IR.

#### Dorsal Striatum Heat Maps

Most of the quantitative differences described above are visible in heat maps of FosB-IR in the DS (Fig. 5), which summarize patterns of labeling in each group. The significantly higher FosB-IR in cocaine-treated rats and the medial-lateral gradient in FosB-IR are visible in the heat maps. While not as prominent as cocaine-saline or medial-lateral differences, higher FosB-IR in females, especially in saline-treated rats, is also evident. Finally, the greater effect of cocaine on FosB-IR in the posterior sections is evident in the heat maps.

**Figure 7:**
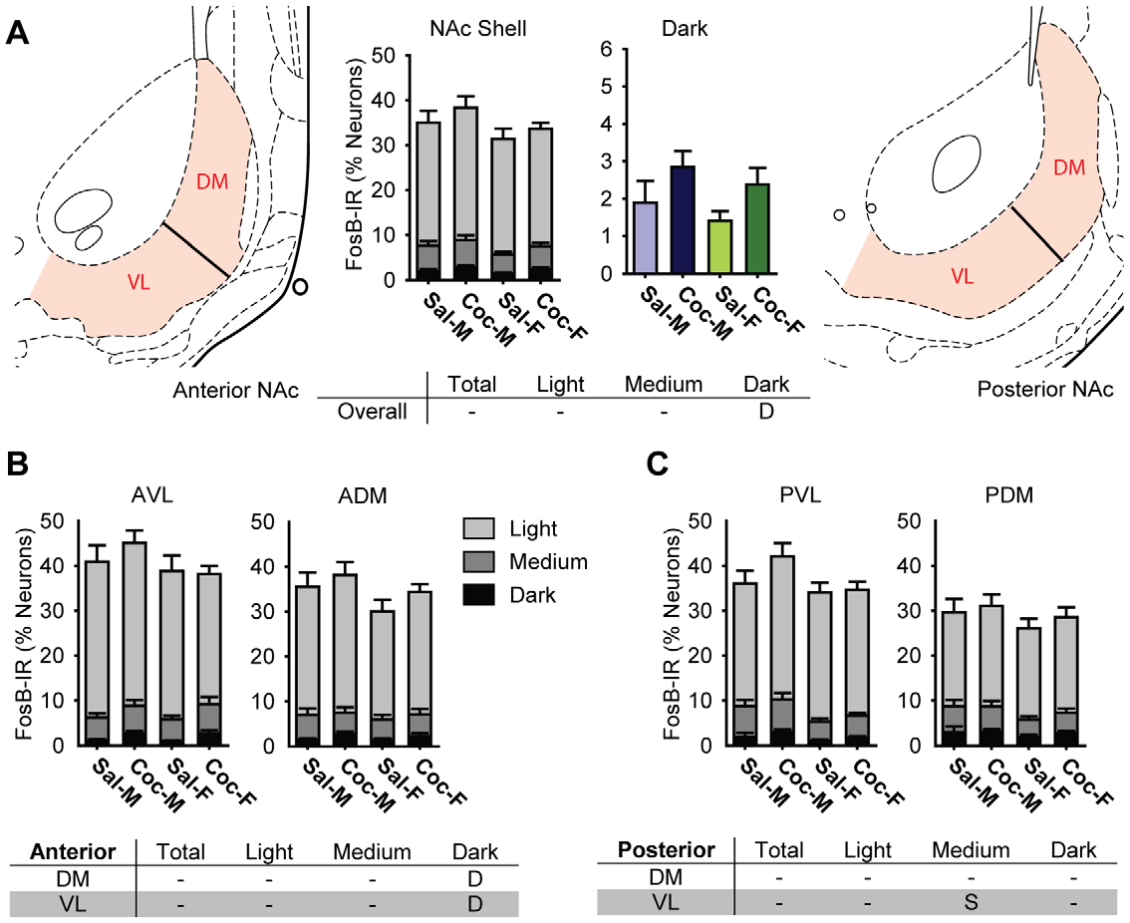
Cocaine selectively increased the proportions of neurons darkly stained for FosB in the nucleus accumbens (NAc) Shell of male and female rats. A) A Bar graphs showing the percentage of all neurons in saline- (Sal) and cocaine- (Coc) treated male (M) and female (F) rats that were FosB-IR. Each bar shows the percentage of all neurons in each treatment group that were light, medium, or darkly stained (middle left, NAc Shell), and darkly stained cells only (middle right, Dark). The partitioning scheme used to define dorsomedial (DM) and ventrolateral (VL) zones for anterior (left) and posterior (right) NAc Shell are also shown. B and C) Bar graphs showing the proportion of FosB-IR cells in each anatomical zone of the anterior (B) and posterior (C) NAc Shell. Each bar shows the percentage of all neurons in each treatment group that were light, medium, or darkly stained. The tables summarize statistically significant differences (p<0.05) for the NAc Shell as a whole (A), in the anterior (B) and the posterior (C) anatomical zones. “D” indicates a statistically significant drug effect, and “S” indicates a significant sex difference (See text).

#### FosB and Locomotor Sensitization: Dorsal Striatum

Comparison of FosB-IR in sensitizing vs. nonsensitizing cocaine-treated rats showed only minor differences. Specifically, the proportion of lightly stained cells differed depending on sex and sensitization classification (sex×sensitization: F_1,13_ = 6.5, p = 0.02). Among nonsensitizing rats, the proportion of lightly stained cells was higher in females than males (males: 17.6±1.4%, and females: 25.0±1.2%, F_1,4_ = 14.4, p = 0.02), whereas there was no sex difference in sensitizing rats (males: 21.5±0.8%, and females: 21.2±0.9%, F_1,8_ = 0.1, ns).

### FosB-IR in the Nucleus Accumbens Core

Overall, proportion of cells that were FosB-IR was higher in the NAc Core (30-50%) than the DS (15–35%). In contrast to the clear effects of cocaine in the DS, however, the effects of cocaine on FosB-IR were smaller in the NAc, including the Core, and were limited to darkly stained cells (Fig. 6A). The proportion of darkly stained cells was 93% higher in cocaine-treated rats than in the saline-treated rats (saline: 2.0±0.2% vs. cocaine: 3.8±0.4%, F_1,55_ = 25.6, p<0.001). There were no drug-induced changes in light or medium stained cells, nor in the proportion of all neuron-like cells that contained FosB-IR. All statistically significant differences in the NAc Core are indicated in the tables in Fig. 6.

#### FosB-IR in the Nucleus Accumbens Core Subregions

The zones used for subregional analyses within the NAc Core are shown in Fig 6A (left: anterior NAc, and right: posterior NAc). The proportions of all neuron-like cells containing FosB-IR in each zone are shown in Fig. 6B (anterior NAc Core) and Fig. 6C (posterior NAc Core). Statistically significant differences in FosB-IR within individual zones of the NAc Core are summarized in Fig. 6B (anterior) and Fig. 6C (posterior). Subregional analyses revealed that, in both the anterior and the posterior NAc Core, cocaine increased the proportion of darkly stained cells in all zones, except for anterior ventrolateral and posterior ventromedial (p = 0.052 and p = 0.15, respectively).

As observed in the DS, we found a significant medial-lateral and anterior-posterior gradients in FosB-IR the NAc Core. The proportion of darkly stained cells was higher in the medial than lateral NAc Core (medial: 3.3±0.3% vs. lateral: 1.9±0.2%, F_1,55_ = 7.0, p = 0.011), and the proportion of darkly stained cells was higher in the posterior than anterior NAc Core (anterior: 2.1±0.2% vs. posterior: 3.2±0.2%, F_3,165_ = 6.3, p<0.001). In addition, there was a dorsal-ventral gradient; the proportion of medium stained cells was higher in the dorsal NAc Core than ventral NAc Core (dorsal: 3.2±0.3% vs. ventral: 2.1±0.3%, F_1,55_ = 4.43, p = 0.04).

#### Sex Differences in Nucleus Accumbens Core FosB-IR

Whereas cocaine-induced differences in FosB-IR were found in the proportions of darkly stained cells, sex differences were found in the proportions of light and medium stained cells, and in the dorsal but not ventral NAc Core (see tables in Fig.6B and 6C). Unlike the DS where females exhibited higher FosB-IR, males exhibited slightly higher FosB-IR in the NAc Core overall, but this was not statistically significant (males: 41.7±1.5% vs. females: 39.0±1.3%, F_1,55_ = 2.0, ns). Considering zones separately, we saw significantly higher proportions of medium (in the anterior dorsolateral, F_1,55_ = 6.6, p = 0.01) and light (in the posterior dorsomedial, F_1,55_ = 4.1, p = 0.048) cells in males than in females. Furthermore, the total proportion of FosB-IR cells was higher in males than in females in anterior dorsal medial zone (F_1,55_ = 4.7, p = 0.03). These sex differences were quite small in magnitude, however, and there were no sex×drug interactions among any of the zones in the Core.

#### Locomotor Sensitization and FosB: Nucleus Accumbens Core

Comparison of FosB-IR in the Core of sensitizing vs. nonensitizing cocaine-treated rats showed a 3-way interaction in the proportion of darkly stained cells (DV x ML x sensitization: F_1,25_5.2, p0.03), but no post-hoc test was statistically significant.

### FosB-IR in the Nucleus Accumbens Shell

The pattern of FosB-IR in the NAc Shell was similar to that in the NAc Core. Namely, there was a cocaine-induced increase in darkly-stained cells (saline: 1.6±0.3% vs. cocaine: 2.6±0.3%, Fig. 7A) and the magnitude of this increase was smaller than in the DS. The proportions of medium or lightly stained cells were not significantly different between groups. All statistically significant differences in the NAc Shell are indicated in the tables in Fig. 7.

#### FosB-IR in the Nucleus Accumbens Shell Subregions

The zones used for subregional analyses are shown in Fig. 7A (left: anterior NAc; right: posterior NAc). The proportions of FosB-IR in each zone are shown in Fig. 7B (anterior NAc Shell) and Fig. 7C (posterior NAc Shell). As was the case in NAc Core, cocaine-induced increases in the proportions of darkly stained cells were found in most zones, but in the shell, these were statistically significant only in the anterior sections (Fig. 7B).

#### Sex Differences in Nucleus Accumbens Shell FosB-IR

As in the NAc Core, males exhibited slightly higher FosB-IR in the NAc shell than females did. This sex difference was significant only in medium stained cells and was apparent only in the posterior sections (Fig. 7C). Males showed significantly higher proportions of medium stained cells in the posterior ventrolateral zone (F_1,55_8.6, p0.005) and a strong trend in the posterior dorsomedial zone (p0.059). In the NAc Shell as a whole, males exhibited higher proportions of FosB-IR cells overall (Fig. 7A; medium, dark, total FosB), but the difference was not statistically significant (medium: F_1,55_ = 0.9, dark: F_1,55_ = 0.9, total FosB: F_1,55_ = 0.8, all ns).

#### Locomotor Sensitization and FosB: Nucleus Accumbens Shell

Similar to analysis in the Core, comparing FosB-IR the Shell of in sensitizing vs. nonsensitizing rats showed a 4-way interaction (AP x zone x sex x sensitization: dark: F_3,75_ = 15.2, p = 0.04 and total FosB: F_3,75_ = 209.9, p = 0.02), but no post-hoc test was statistically significant.

#### Nucleus Accumbens Heat Maps

**Figure 8:**
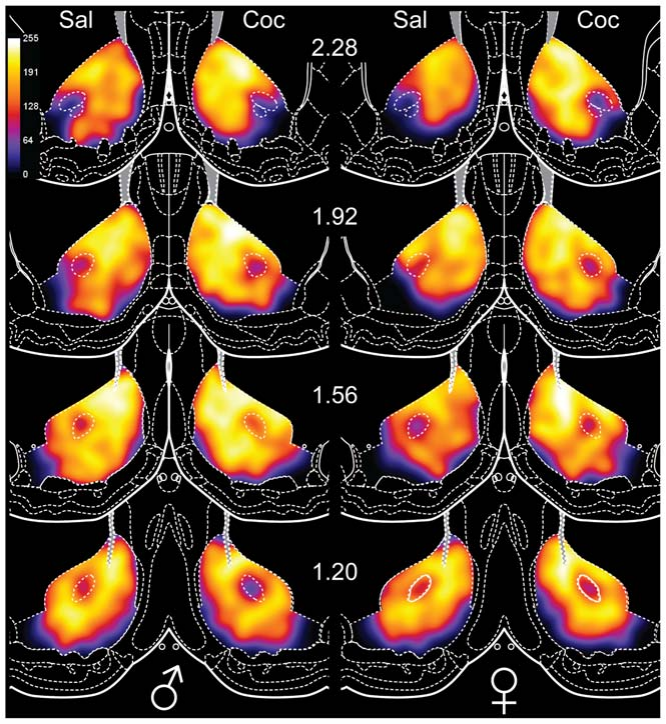
Heat maps of FosB-IR in the nucleus accumbens. Numbers in the center indicate AP levels relative to Bregma. Unlike the dorsal striatum, the cocaine-induced increase in FosB-IR is relatively small for both males (left) and females (right). Particularly high FosB-IR near the lateral ventricle is evident in most maps. Males exhibited higher FosB-IR, which is more apparent in the saline-treated rats. In addition, medial-lateral, as well as dorsal-ventral gradients in FosB-IR are visible at most levels. Coc: cocaine, and Sal: saline.

Heat maps displaying FosB-IR in the NAc of saline- vs. cocaine-treated rats (Fig. 8) show few differences overall, consistent with the relatively small effects of cocaine and sex differences in FosB-IR in the NAc. Nonetheless, medial-lateral and dorsal-ventral gradients in FosB-IR are visible. The heat maps also show subregional variation in FosB-IR, with the greatest proportion of FosB-IR cells in the dorsomedial core, adjacent to the lateral ventricle. Additionally, the generally higher level of FosB-IR in the NAc compared to the DS is apparent, particularly in the controls (compare Fig. 5 and Fig. 8).

## Discussion

Repeated exposure to psychostimulants causes long-term changes in the brain. Previous studies have demonstrated the importance of ΔFosB in these changes [reviewed in 1]. In the current study, we provide a detailed quantitative analysis of cocaine-induced ΔFosB accumulation in subregions of the dorsal striatum and nucleus accumbens in behaviorally characterized male and female rats. As others have found [44], [45], females showed significantly greater locomotor activity in response to cocaine than males. Interestingly, however, breaking down the data by sensitizing and nonsensitizing rats showed that this was due largely to sex differences among nonsensitizing rats. Nonsensitizing males showed a low initial response to cocaine that stayed low throughout treatment, whereas nonsensitizing females showed a high initial response that stayed high. The behavior of sensitizing males and females was quite similar except that maximal locomotor activity was higher in females.

We identified 5G4 anti-FosB as being slightly better than sc-48 and much better than H-75 for recognizing ΔFosB isoforms. Using 5G4, we found that cocaine clearly increased total ΔFosB levels in the DS and NAc, as assessed by western blot. The cocaine-induced increase was greater in the DS than in the NAc, as previously shown [46]. Mapping ΔFosB positive cells showed that basal ΔFosB levels were highest in the medial DS and the dorsomedial NAc, areas adjacent to the lateral ventricles, but that the largest cocaine-induced changes were in the lateral DS, where basal ΔFosB levels were low. Overall, ΔFosB was expressed in up to half of the neurons in the striatum (10–20% in low areas vs. ∼50% in high areas), consistent with the proportion of striatonigral cells in which ΔFosB is preferentially expressed [13], [14]. The effect of cocaine was primarily to increase the intensity of ΔFosB immunostaining rather than to increase the proportion of cells with ΔFosB positive nuclei. Namely, the overall proportions of cells containing any FosB-IR did not change in most areas, while the proportion of heavily stained cells increased with cocaine-treatment. Sex differences in ΔFosB accumulation were small and regionally specific; DS levels were higher in females whereas NAc levels were higher in males. Cocaine-induced increases in ΔFosB and these small sex differences were additive, as no interactions were observed.

### Sex differences in psychostimulant-induced locomotion

One interesting finding from our behavioral analysis was that the wide range of initial responses to cocaine in females was closely linked to subsequent development of sensitization. Understanding the factors that lead to locomotor sensitization is important because there is evidence that the changes underlying locomotor sensitization also mediate potentiation of drug taking following prior drug exposure [see 47 for review]. The multiple drug treatments typically used to assess sensitization are known to disrupt the estrous cycle [reviewed in 48] and likely cause brain changes on their own, masking pre-existing differences. Thus being able to differentiate between females that would go on to show sensitization (or not) using a single drug treatment could be useful to identify pre-existing differences that predict sensitization or the lack of sensitization.

One factor uncontrolled in the current study that may have significant influence is the hormonal status of females, as estrogens have been shown to enhance the locomotor-activating effects of psychostimulants [reviewed in 16,17,29] and the rate of sensitization in females [49]. However, it is unlikely that the wider range of initial responses in females that we saw was due solely to their estrous phase, as Walker et al., [45] have shown that even ovariectomized females show a much wider range of initial responses than males. In addition, that sensitization was similar between males and females in our study argues against a major role for estrogens in modulating sensitization of locomotor behavior. Others have also suggested that individual differences in vulnerability to sensitization and hormonal influences are independent and can be dissociated [19], [50]. It remains possible, however, that hormonal modulation of behavioral responses to psychostimulants depends on what type of behavior is assayed (e.g., locomotor behavior vs. rotational behavior).

In contrast to the wide range of behavioral responses observed upon the initial injection in females, males showed a wider spread of behavior during the second week. This pattern is reminiscent of the bimodal distribution of behavior and associated differences in dopaminergic activity observed only in males following repeated psychostimulant injections [19]. Thus, perhaps females have a wider range of pre-existing differences that affect initial responses to psychostimulants, while males have more diverse psychostimulant-induced responses to repeated treatment.

While there were significant differences in ΔFosB accumulation between saline- and cocaine-treated animals, we found no clear relationship between locomotor sensitization and ΔFosB accumulation in the DS or NAc within the animals treated with cocaine. This observation is consistent with Kelz, et al., [2] in which overexpression of ΔFosB potentiated cocaine-induced locomotion but did not affect locomotor sensitization. It is possible that the design of the current study, with a single cocaine dose, did not produce a sufficient range of behavior or ΔFosB accumulation to detect such a relationship. Alternatively, it could be that accumulation of ΔFosB beyond a certain threshold level is necessary to trigger changes associated with locomotor sensitization. Because there likely are multiple steps between ΔFosB accumulation and behavioral output, other intervening factors may be more closely associated with locomotor sensitization. For example, we have recently reported a correlation between locomotor behavior and MSN spine density in the NAc [51].

### Sex differences in ΔFosB accumulation

We found that sex differences in ΔFosB accumulation are small and largely independent of cocaine-treatment, likely reflecting pre-existing differences rather than differential responses to cocaine. In the DS, sex differences were consistent with the behavioral differences: females showed greater locomotor activation and higher ΔFosB levels than males. In contrast, in the NAc, males showed higher ΔFosB levels. Within the subregions of the DS and NAc, sex differences were generally small and non-significant in most cases. The sex differences in ΔFosB levels independent of cocaine-treatment are consistent with sex differences in locomotion upon the initial exposure to psychostimulants in the current and previous studies [22], [44], [45].

### Subregional variation in ΔFosB expression

The NAc is strongly implicated in locomotion, while the DS is more closely associated with stereotypy [52]. As previously mentioned, we saw no clear relationship between locomotor sensitization and ΔFosB in either region, at least within cocaine treated animals. Further studies are required to determine if individual variations in cocaine-induced ΔFosB accumulation in specific striatal subregions are associated with other cocaine-induced behaviors, including stereotypy. Additionally, cocaine was experimenter administered in this study, thus it is unknown how regional specificity of ΔFosB may be influenced by self-administered cocaine. Nonetheless, the regionally specific patterns of ΔFosB accumulation that we observed parallel known differences in afferents to striatal subregions [53]. Because ΔFosB expression in MSNs requires both dopaminergic and glutamatergic stimulation [2], [31], [54], [55], differences in one or both types of input could correspond to variation in basal ΔFosB levels and accumulation in response to cocaine. In turn, such differences in connectivity are related to the involvement of striatal subregions in different aspects of behavior.

Striatal regions with high basal ΔFosB-IR, the NAc Core and the medial DS, receive dopaminergic inputs mainly from the lateral ventral tegmental area and the medial substantia nigra pars compacta (SNc) [56, reviewed in 57]. Glutamatergic inputs come from medial prefrontal cortex (prelimbic and infralimbic areas) [58], subiculum [59], basolateral amygdala [60], [61], as well as midline and medial intralaminar thalamic nuclei [62]. In contrast, the lateral DS, which had low basal ΔFosB-IR but showed the greatest effects of cocaine, receives dopaminergic inputs from the lateral SNc [56, reviewed in 57] and glutamatergic inputs from somatosensory cortex [58] and lateral intralaminar thalamic nuclei [62]. The medial DS has been implicated in goal-directed behaviors, while the lateral DS is involved in habit formation [see 63 for review,64].

In the context of drug abuse, functional differences between striatal subregions have been linked to acquisition of operant self-administration (medial DS) and its eventual transition to habitual responding that is resistant to reward devaluation (lateral DS) [65]. Effects in the goal-directed circuit observed in the current study likely contribute to the potentiation of drug taking by prior drug exposure [reviewed in 47]. The even larger changes we found in the habit circuit suggest that the potentiation of habit formation by prior exposure to drug [see 66 for example] may be more pronounced than the potentiation of drug taking, which could facilitate transition to habitual drug taking.

### ΔFosB, MAPK, and structural plasticity of MSNs

ΔFosB is closely linked to psychostimulant-induced changes in MSN dendritic spine density [14], which is thought to reflect rewiring of reward circuitry related to addiction. Psychostimulant-induced structural plasticity of MSN dendrites also requires activation of the MAPK signaling pathway [67], [68], [69] and MAPK is important in the development of behavioral sensitization to psychostimulants [70], [71]. Indeed, the pattern of ΔFosB accumulation we observed corresponds closely with other studies of cocaine-induced MAPK activation [30], [70], as demonstrated by ERK phosphorylation.

Given the relationship between ΔFosB and downstream consequences of psychostimulant exposure, striatal subregions with more pronounced cocaine-induced ΔFosB accumulation might be expected to show more robust drug-induced structural changes. Interestingly, Jedynak, et al. [72] found that repeated methamphetamine injections increase MSN spine density in the lateral DS, where we saw the greatest cocaine effect on ΔFosB, while decreasing spine density in the medial DS, where we saw the smallest effect on ΔFosB. Thus our findings may reflect subregional variation in the underlying processes leading to psychostimulant-induced rewiring of neural circuits.

### Conclusion

Our results suggest that sex differences in psychostimulant-induced locomotion and ΔFosB accumulation are more likely due to pre-existing differences between males and females rather than to sex-specific responses to repeated psychostimulant exposure. Additionally, our behavioral analyses indicate that, especially in females, the locomotor response upon initial exposure to psychostimulants may be useful as a proxy for subsequent development of sensitization. It has been argued that locomotor sensitization shares underlying mechanisms with other behavioral changes related to drug addiction [47], [73]. As such, responses to an initial cocaine exposure may help to identify pre-existing individual differences associated with susceptibility to addiction.

The regionally specific patterns of ΔFosB accumulation observed in this study add to the existing literature on the heterogeneous nature of striatal responses to psychostimulants. The medial and the lateral DS exhibited a striking difference in ΔFosB accumulation, which is consistent with functional and anatomical differences between these subregions. Thus, distinguishing between medial and lateral DS in future studies, rather than treating them as a single structure, should benefit further elucidation of the how the DS is involved in drug abuse, especially in the transition to habitual drug taking.

## References

[1] Nestler EJ (2008). Review. Transcriptional mechanisms of addiction: role of DeltaFosB.. Philos Trans R Soc Lond B Biol Sci.

[2] Kelz MB, Chen J, Carlezon WA, Whisler K, Gilden L (1999). Expression of the transcription factor deltaFosB in the brain controls sensitivity to cocaine.. Nature.

[3] Colby CR, Whisler K, Steffen C, Nestler EJ, Self DW (2003). Striatal cell type-specific overexpression of DeltaFosB enhances incentive for cocaine.. J Neurosci.

[4] Hope B, Kosofsky B, Hyman SE, Nestler EJ (1992). Regulation of immediate early gene expression and AP-1 binding in the rat nucleus accumbens by chronic cocaine.. Proc Natl Acad Sci U S A.

[5] Groenewegen HJ, Wright CI, Beijer AV, Voorn P (1999). Convergence and segregation of ventral striatal inputs and outputs.. Ann N Y Acad Sci.

[6] Zaborszky L, Pang K, Somogyi J, Nadasdy Z, Kallo I (1999). The basal forebrain corticopetal system revisited.. Ann N Y Acad Sci.

[7] Zahm DS (1999). Functional-anatomical implications of the nucleus accumbens core and shell subterritories.. Ann N Y Acad Sci.

[8] Di Chiara G (2002). Nucleus accumbens shell and core dopamine: differential role in behavior and addiction.. Behav Brain Res.

[9] Gerfen CR (1992). The neostriatal mosaic: multiple levels of compartmental organization in the basal ganglia.. Annu Rev Neurosci.

[10] Gerfen CR, Engber TM, Mahan LC, Susel Z, Chase TN (1990). D1 and D2 dopamine receptor-regulated gene expression of striatonigral and striatopallidal neurons.. Science.

[11] Le Moine C, Bloch B (1995). D1 and D2 dopamine receptor gene expression in the rat striatum: sensitive cRNA probes demonstrate prominent segregation of D1 and D2 mRNAs in distinct neuronal populations of the dorsal and ventral striatum.. J Comp Neurol.

[12] Lu XY, Ghasemzadeh MB, Kalivas PW (1998). Expression of D1 receptor, D2 receptor, substance P and enkephalin messenger RNAs in the neurons projecting from the nucleus accumbens.. Neuroscience.

[13] Moratalla R, Elibol B, Vallejo M, Graybiel AM (1996). Network-level changes in expression of inducible Fos-Jun proteins in the striatum during chronic cocaine treatment and withdrawal.. Neuron.

[14] Lee KW, Kim Y, Kim AM, Helmin K, Nairn AC (2006). Cocaine-induced dendritic spine formation in D1 and D2 dopamine receptor-containing medium spiny neurons in nucleus accumbens.. Proc Natl Acad Sci U S A.

[15] Perrotti LI, Weaver RR, Robison B, Renthal W, Maze I (2008). Distinct patterns of DeltaFosB induction in brain by drugs of abuse.. Synapse.

[16] Becker JB, Hu M (2008). Sex differences in drug abuse.. Front Neuroendocrinol.

[17] Lynch WJ, Roth ME, Carroll ME (2002). Biological basis of sex differences in drug abuse: preclinical and clinical studies.. Psychopharmacology (Berl).

[18] Roth ME, Cosgrove KP, Carroll ME (2004). Sex differences in the vulnerability to drug abuse: a review of preclinical studies.. Neurosci Biobehav Rev.

[19] Camp DM, Robinson TE (1988). Susceptibility to sensitization. I. Sex differences in the enduring effects of chronic D-amphetamine treatment on locomotion, stereotyped behavior and brain monoamines.. Behav Brain Res.

[20] Castner SA, Xiao L, Becker JB (1993). Sex differences in striatal dopamine: in vivo microdialysis and behavioral studies.. Brain Res.

[21] Peris J, Decambre N, Coleman-Hardee ML, Simpkins JW (1991). Estradiol enhances behavioral sensitization to cocaine and amphetamine-stimulated striatal [3H]dopamine release.. Brain Res.

[22] Robinson TE, Becker JB, Ramirez VD (1980). Sex differences in amphetamine-elicited rotational behavior and the lateralization of striatal dopamine in rats.. Brain Res Bull.

[23] Lynch WJ, Carroll ME (1999). Sex differences in the acquisition of intravenously self-administered cocaine and heroin in rats.. Psychopharmacology (Berl).

[24] Roberts DC, Bennett SA, Vickers GJ (1989). The estrous cycle affects cocaine self-administration on a progressive ratio schedule in rats.. Psychopharmacology (Berl).

[25] Russo SJ, Jenab S, Fabian SJ, Festa ED, Kemen LM (2003). Sex differences in the conditioned rewarding effects of cocaine.. Brain Res.

[26] Hu M, Watson CJ, Kennedy RT, Becker JB (2006). Estradiol attenuates the K+-induced increase in extracellular GABA in rat striatum.. Synapse.

[27] Mermelstein PG, Becker JB, Surmeier DJ (1996). Estradiol reduces calcium currents in rat neostriatal neurons via a membrane receptor.. J Neurosci.

[28] Forlano PM, Woolley CS (2010). Quantitative analysis of pre- and postsynaptic sex differences in the nucleus accumbens.. J Comp Neurol.

[29] Becker JB (1999). Gender differences in dopaminergic function in striatum and nucleus accumbens.. Pharmacol Biochem Behav.

[30] Radwanska K, Valjent E, Trzaskos J, Caboche J, Kaczmarek L (2006). Regulation of cocaine-induced activator protein 1 transcription factors by the extracellular signal-regulated kinase pathway.. Neuroscience.

[31] Zhang D, Zhang L, Lou DW, Nakabeppu Y, Zhang J (2002). The dopamine D1 receptor is a critical mediator for cocaine-induced gene expression.. J Neurochem.

[32] Harlan RE, Garcia MM (1998). Drugs of abuse and immediate-early genes in the forebrain.. Mol Neurobiol.

[33] Castner SA, Becker JB (1996). Sex differences in the effect of amphetamine on immediate early gene expression in the rat dorsal striatum.. Brain Res.

[34] Nestler EJ, Barrot M, Self DW (2001). DeltaFosB: a sustained molecular switch for addiction.. Proc Natl Acad Sci U S A.

[35] Perrotti LI, Hadeishi Y, Ulery PG, Barrot M, Monteggia L (2004). Induction of deltaFosB in reward-related brain structures after chronic stress.. J Neurosci.

[36] Jiao Y, Sun Z, Lee T, Fusco FR, Kimble TD (1999). A simple and sensitive antigen retrieval method for free-floating and slide-mounted tissue sections.. J Neurosci Methods.

[37] Hart SA, Patton JD, Woolley CS (2001). Quantitative analysis of ER alpha and GAD colocalization in the hippocampus of the adult female rat.. J Comp Neurol.

[38] Gundersen HJ, Jensen EB (1987). The efficiency of systematic sampling in stereology and its prediction.. J Microsc.

[39] West MJ, Slomianka L, Gundersen HJ (1991). Unbiased stereological estimation of the total number of neurons in thesubdivisions of the rat hippocampus using the optical fractionator.. Anat Rec.

[40] Gundersen HJ, Jensen EB, Kieu K, Nielsen J (1999). The efficiency of systematic sampling in stereology--reconsidered.. J Microsc.

[41] Steiner H, Gerfen CR (1993). Cocaine-induced c-fos messenger RNA is inversely related to dynorphin expression in striatum.. J Neurosci.

[42] Paxinos G, Watson C (2004). The Rat Brain: in sterotaxic coordinates, 5th ed..

[43] Boudreau AC, Wolf ME (2005). Behavioral sensitization to cocaine is associated with increased AMPA receptor surface expression in the nucleus accumbens.. J Neurosci.

[44] Festa ED, Russo SJ, Gazi FM, Niyomchai T, Kemen LM (2004). Sex differences in cocaine-induced behavioral responses, pharmacokinetics, and monoamine levels.. Neuropharmacology.

[45] Walker QD, Cabassa J, Kaplan KA, Li ST, Haroon J (2001). Sex differences in cocaine-stimulated motor behavior: disparate effects of gonadectomy.. Neuropsychopharmacology.

[46] Larson EB, Akkentli F, Edwards S, Graham DL, Simmons DL (2010). Striatal regulation of DeltaFosB, FosB, and cFos during cocaine self-administration and withdrawal.. J Neurochem.

[47] Vezina P (2004). Sensitization of midbrain dopamine neuron reactivity and the self-administration of psychomotor stimulant drugs.. Neurosci Biobehav Rev.

[48] Mello NK, Mendelson JH (1997). Cocaine's effects on neuroendocrine systems: clinical and preclinical studies.. Pharmacol Biochem Behav.

[49] Hu M, Becker JB (2003). Effects of sex and estrogen on behavioral sensitization to cocaine in rats.. J Neurosci.

[50] Robinson TE (1984). Behavioral sensitization: characterization of enduring changes in rotational behavior produced by intermittent injections of amphetamine in male and female rats.. Psychopharmacology (Berl).

[51] Wissman AM, McCollum AF, Huang GZ, Nikrodhanond AA, Woolley CS (2011). Sex differences and effects of cocaine on excitatory synapses in the nucleus accumbens..

[52] Delfs JM, Schreiber L, Kelley AE (1990). Microinjection of cocaine into the nucleus accumbens elicits locomotor activation in the rat.. J Neurosci.

[53] Voorn P, Vanderschuren LJ, Groenewegen HJ, Robbins TW, Pennartz CM (2004). Putting a spin on the dorsal-ventral divide of the striatum.. Trends Neurosci.

[54] Bronstein DM, Ye H, Pennypacker KR, Hudson PM, Hong JS (1994). Role of a 35 kDa fos-related antigen (FRA) in the long-term induction of striatal dynorphin expression in the 6-hydroxydopamine lesioned rat.. Brain Res Mol Brain Res.

[55] Nye HE, Hope BT, Kelz MB, Iadarola M, Nestler EJ (1995). Pharmacological studies of the regulation of chronic FOS-related antigen induction by cocaine in the striatum and nucleus accumbens.. J Pharmacol Exp Ther.

[56] Fallon JH, Moore RY (1978). Catecholamine innervation of the basal forebrain. IV. Topography of the dopamine projection to the basal forebrain and neostriatum.. J Comp Neurol.

[57] Joel D, Weiner I (2000). The connections of the dopaminergic system with the striatum in rats and primates: an analysis with respect to the functional and compartmental organization of the striatum.. Neuroscience.

[58] McGeorge AJ, Faull RL (1989). The organization of the projection from the cerebral cortex to the striatum in the rat.. Neuroscience.

[59] Brog JS, Salyapongse A, Deutch AY, Zahm DS (1993). The patterns of afferent innervation of the core and shell in the "accumbens" part of the rat ventral striatum: immunohistochemical detection of retrogradely transported fluoro-gold.. J Comp Neurol.

[60] Kita H, Kitai ST (1990). Amygdaloid projections to the frontal cortex and the striatum in the rat.. J Comp Neurol.

[61] Wright CI, Beijer AV, Groenewegen HJ (1996). Basal amygdaloid complex afferents to the rat nucleus accumbens are compartmentally organized.. J Neurosci.

[62] Berendse HW, Groenewegen HJ (1990). Organization of the thalamostriatal projections in the rat, with special emphasis on the ventral striatum.. J Comp Neurol.

[63] Balleine BW, Liljeholm M, Ostlund SB (2009). The integrative function of the basal ganglia in instrumental conditioning.. Behav Brain Res.

[64] Graybiel AM (2008). Habits, rituals, and the evaluative brain.. Annu Rev Neurosci.

[65] Robbins TW, Ersche KD, Everitt BJ (2008). Drug addiction and the memory systems of the brain.. Ann N Y Acad Sci.

[66] Nelson A, Killcross S (2006). Amphetamine exposure enhances habit formation.. J Neurosci.

[67] Girault JA, Valjent E, Caboche J, Herve D (2007). ERK2: a logical AND gate critical for drug-induced plasticity?. Curr Opin Pharmacol.

[68] Thomas GM, Huganir RL (2004). MAPK cascade signalling and synaptic plasticity.. Nat Rev Neurosci.

[69] Russo SJ, Dietz DM, Dumitriu D, Morrison JH, Malenka RC (2010). The addicted synapse: mechanisms of synaptic and structural plasticity in nucleus accumbens.. Trends Neurosci.

[70] Valjent E, Bertran-Gonzalez J, Aubier B, Greengard P, Herve D (2010). Mechanisms of locomotor sensitization to drugs of abuse in a two-injection protocol.. Neuropsychopharmacology.

[71] Valjent E, Pages C, Herve D, Girault JA, Caboche J (2004). Addictive and non-addictive drugs induce distinct and specific patterns of ERK activation in mouse brain.. Eur J Neurosci.

[72] Jedynak JP, Uslaner JM, Esteban JA, Robinson TE (2007). Methamphetamine-induced structural plasticity in the dorsal striatum.. Eur J Neurosci.

[73] Vanderschuren LJ, Kalivas PW (2000). Alterations in dopaminergic and glutamatergic transmission in the induction and expression of behavioral sensitization: a critical review of preclinical studies.. Psychopharmacology (Berl).

